# Neuromorphic Computing Using NAND Flash Memory Architecture With Pulse Width Modulation Scheme

**DOI:** 10.3389/fnins.2020.571292

**Published:** 2020-09-18

**Authors:** Sung-Tae Lee, Jong-Ho Lee

**Affiliations:** Department of Electrical and Computer Engineering, ISRC, Seoul National University, Seoul, South Korea

**Keywords:** neuromorphic, synaptic device, in-memory computing, NAND flash, deep neural networks, quantized neural networks

## Abstract

A novel operation scheme is proposed for high-density and highly robust neuromorphic computing based on NAND flash memory architecture. Analog input is represented with time-encoded input pulse by pulse width modulation (PWM) circuit, and 4-bit synaptic weight is represented with adjustable conductance of NAND cells. Pulse width modulation scheme for analog input value and proposed operation scheme is suitably applicable to the conventional NAND flash architecture to implement a neuromorphic system without additional change of memory architecture. Saturated current-voltage characteristic of NAND cells eliminates the effect of serial resistance of adjacent cells where a pass bias is applied in a synaptic string and IR drop of metal wire resistance. Multiply–accumulate (MAC) operation of 4-bit weight and width-modulated input can be performed in a single input step without additional logic operation. Furthermore, the effect of quantization training (QT) on the classification accuracy is investigated compared with post-training quantization (PTQ) with 4-bit weight. Lastly, a sufficiently low current variance of NAND cells obtained by the read–verify–write (RVW) scheme achieves satisfying accuracies of 98.14 and 89.6% for the MNIST and CIFAR10 images, respectively.

## Introduction

Recently, deep neural networks (DNNs) have achieved excellent performance for a variety of intelligent tasks, such as natural language processing, computer vision, and speech recognition (Truong et al., 2016; Nishani and Cico, 2017; Sainath et al., 2017). However, recent high-performance DNNs require a vast network size and an enormous number of parameters and computational capability, which demand very fast and power-hungry graphics processing units (Scardapane et al., 2017; Khan et al., 2019). Furthermore, von Neumann architecture leads to tremendous time and energy consumption due to the bottleneck between memory and processor. To accelerate neural network computation, neuromorphic systems that can efficiently process multiply–accumulate (MAC) operation have been proposed and developed utilizing memory devices (Suri et al., 2011; Jackson et al., 2013).

In prior research, resistive random access memories (RRAMs) were mainly used as synaptic devices to implement the neuromorphic system (Park et al., 2013; Tang et al., 2017; Andri et al., 2018; Zhou et al., 2018; Guan and Ohsawa, 2019). However, RRAMs require further research in terms of cell characteristics variation, reliability, and integration of selectors for large-scale integration (Woo and Yu, 2019). In addition, the effect of metal wire resistance can cause inaccurate vector–matrix multiplication (VMM) operation in a large array (Wang et al., 2020). Furthermore, low on/off current ratio of RRAMs restricts bandwidth to sum current of many RRAM devices (Sun et al., 2018; Yu et al., 2020). The state-of-the-art algorithms typically demand a huge parameter size. To satisfy this demand, NAND flash memory can be a promising candidate for a synaptic device to meet this requirement. NAND flash memory offers ultra-high bit density for immense data storage and low fabrication cost per bit, and it has been well known as a mature technology (Yamashita et al., 2017; Kang et al., 2019; Huh et al., 2020). However, NAND flash memory was not commonly used in neuromorphic system because of the characteristics of the string structure. In RRAM crossbar array, the input bias is applied to word-lines (WLs), and output current is summed through bit-lines (BLs). Therefore, VMM of the input voltage applied to the WLs and the conductance of the RRAM can be easily implemented. However, in NAND flash memory architecture, the WL and source-line (SL) are shared by NAND strings in the same block. Furthermore, read bias and pass bias are applied to the selected layer and unselected layers, respectively, to read the current of NAND cells of a selected layer. Therefore, it has been considered difficult to implement VMM in NAND flash memory architecture.

In this article, a novel neuromorphic architecture is proposed for the quantized neural network (QNN) utilizing NAND flash memory with a pulse width modulation (PWM) scheme. Our scheme implements a high-density neuromorphic system because two NAND cells having eight current levels (3-bit) are used as one synaptic device, and a PWM circuitry can represent the analog input values. Furthermore, our scheme can process MAC of the analog input value and 4-bit weight with only a single input step, which considerably decreases power consumption and burden of peripheral circuits needed in architectures in digital design. Utilizing saturated current-voltage characteristics of NAND cells solves the problem arising from the resistance of the pass cells where a pass bias is applied and metal wire. Furthermore, the effect of quantization training (QT) on inference accuracy is investigated compared with post-training quantization (PTQ). Lastly, we show that sufficiently low current variance of synaptic devices obtained by the read–verify–write (RVW) method achieves satisfying accuracy.

## Materials and Methods

### Neuromorphic System Using NAND Flash

**FIGURE 1:**
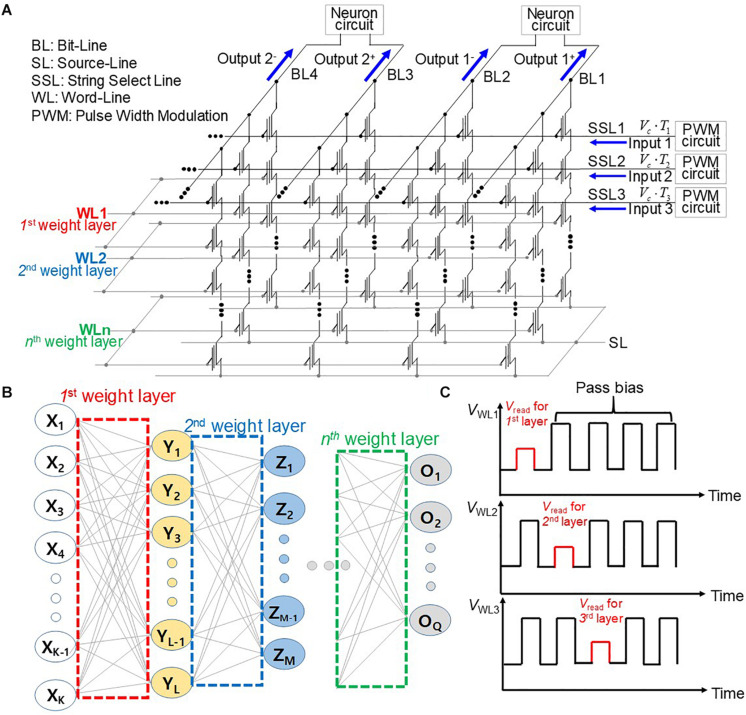
**(A)** Operation scheme for synaptic string array utilizing NAND flash memory with PWM circuits. **(B)** Schematic diagram of neural networks. **(C)** Pulse diagram applied to WLs with the time.

Figure 1 shows schematically an operation scheme of a neuromorphic system utilizing a three-dimensional (3D) NAND flash with PWM circuits. Input voltages with adjustable pulse width from PWM circuits are imposed on string-select lines (SSL), where cell current is added in the BLs, as shown in Figure 1A. The NAND cells in the *k*^th^ WL represent the synapses in the *k*^th^ synaptic layer of the neural network shown in Figure 1B. The read bias (*V*_read_) and pass bias (*V*_PASS_) are imposed on a selected WL and unselected WLs, respectively, as shown in Figure 1C. When *V*_read_ is imposed on the WL sequentially along the synaptic string, the output of each postsynaptic neuron is sequentially generated. Cells are connected to a selected WL store weights, and each weight determines the string-current of each string. In the proposed scheme, the input voltage is simultaneously imposed on all SSLs. The proposed operation scheme is different from that of the conventional NAND flash memory architecture, as compared in Table 1. The input bias corresponding to neuron activation is applied to SSLs, and the current sum is read through BLs in the proposed operation scheme. On the other hand, the cell selected by the input address is read through BL in the conventional NAND flash memory. Furthermore, SSLs are simultaneously biased by input voltage in the proposed scheme, whereas read bias is imposed sequentially on each SSL in the conventional NAND flash memory. Therefore, this scheme significantly reduces latency compared with conventional NAND flash memory technology. The output current is read through the BL in both schemes. In addition, the proposed synaptic architecture utilizing NAND flash is different from the RRAM crossbar array. In the RRAM crossbar array, the input bias is applied to WLs, and the output current is summed through BLs. The NAND cell array is composed of cell strings, and each cell string has multiple cells connected in series. In the NAND cell array, the WL and SL are shared by NAND strings in the same block of NAND flash memory. In addition, to turn on unselected cells, pass bias (*V*_PASS_) should be applied to WLs of unselected cells. Therefore, in the proposed synaptic architecture, the input is applied to SSLs, and the output current is read in the BLs. Furthermore, cells in the *k*^th^ layer in NAND flash strings represent synapses in the *k*^th^ layer synapse layer in neural networks. Note that the proposed operation scheme can be applied to both 2D and 3D NAND flash memory architectures.

**TABLE 1:** Comparison of proposed operation scheme with that of conventional NAND flash memory.

	Proposed operation scheme	Conventional NAND flash memory
Input	Input bias corresponding to neuron activation	Address of a selected cell
String select line	Simultaneously biased	Sequentially read
Output current	Bit-line	Bit-line

**FIGURE 2:**
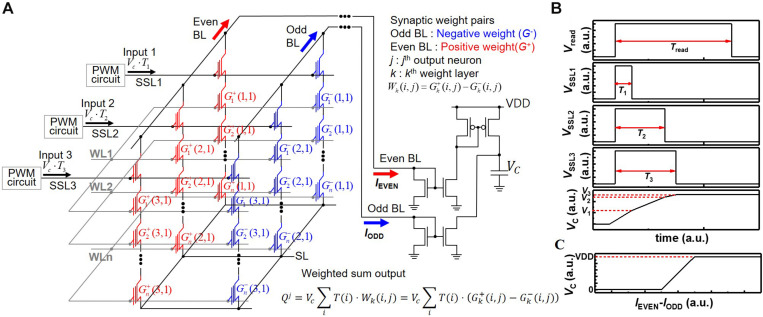
**(A)** Schematic diagram of synaptic string array consisting of synapses with positive weight (*G*^+^) and synapses with negative weight (*G*^–^). **(B)** Pulse diagram of operation scheme and voltage of capacitor with the time. **(C)** Voltage of capacitor (*V*_*C*_) with difference of *I*_*EVEN*_ and *I*_*ODD*_.

Figure 2 represents VMM operation utilizing a string array and neuron circuits. In the neuromorphic system, the weight and input in the DNN algorithm are represented by conductance and input voltage of synaptic devices, respectively. In the DNN algorithm, weighted sum output is linearly increased with input as shown in the equation;

(1) O=∑WX

where *O*, *W*, and *X* represent weighted sum output, weight, and input, respectively. In the neuromorphic system, it is commonly assumed synaptic devices have linear current (*I*) versus voltage (*V*) characteristics (Kim T. et al., 2017). If synaptic devices have linear *I-V* characteristics, the amplitude of input in a DNN model can be simply represented by the amplitude of input voltage of synaptic devices. Then, the weighted sum current is represented by the product of input voltage and conductance of synaptic devices, as shown in the equation;

(2) I=∑GV

where *I*, *G*, and *V* represent weighted sum current, conductance, and input voltage of devices, respectively. On the other hand, the cell device of NAND flash memory has non-linear *I-V* characteristics (Lee et al., 2018, 2019a), which means output current has a non-linear relationship with the input voltage. Thus, an analog amplitude of input pulse cannot represent the amplitude of input in a DNN algorithm (Lee et al., 2019b). To resolve the problem of the non-linear *I-V* characteristic of NAND cells, the PWM scheme is proposed. In this scheme, the amplitude of the input pulse is fixed, whereas the pulse width of the input pulse varies in proportional to the amplitude of input in a DNN algorithm. Then, the weighted sum output is represented by the amount of charge accumulated in neuron circuits, whereas the input voltage is applied as shown in the equation;

(3) Q=V⁢∑GT

where *Q*, *V*, *G*, and *T* represent weighted sum charge, the constant amplitude of input pulse, conductance of device, and pulse width of the input pulse, respectively. Therefore, the weighted sum in a DNN model can be correctly performed in neuromorphic systems by using the PWM scheme despite the non-linear *I-V* characteristics of cell devices. In addition, this scheme is well fitted to conventional NAND flash memory architecture. Two adjacent NAND cells are used for one synaptic device to represent negative weight value. Considering the negative weight, the charge accumulated in the neuron circuit can be represented by the equation;

(4) $$Q=V\,\sum T\,\left(G^{+}-G^{-}\right)$$

where *G*^–^ and *G*^+^ represent negative and positive weights, respectively.

By adopting two current mirrors and one capacitor as one neuron circuit shown in Figure 2A, current summing in time scale and subtracting between positive and negative weights are carried out (Kim H. et al., 2017). In Figure 2A, synaptic devices connected to even BL and odd BL have positive weight (*G*^+^) and negative weight (*G*^–^), respectively. The *k*, *j*, and *i* in the weighted sum equation of Figure 2A represent the *k*^th^ synapse layer, *j*^th^ postsynaptic neuron, and *i*^th^ synapse connected to *j*^th^ neuron, respectively. The current of even BL (*I*_EVEN_) accumulates the charge in a capacitor, and the current of odd BL (*I*_ODD_) reduces the charge in a capacitor. Figure 2B represents the pulse diagram of the operation scheme and voltage of the capacitor (*V*_C_) in the case of positive weight as an example. Whereas *V*_read_ is applied to selected WL during *T*_read_, the *V*_SSL__1_, *V*_SSL__2_, and *V*_SSL__3_ are applied to SSL1, SSL2, and SSL3 during *T*_1_, *T*_2_, and *T*_3_, respectively. Then, *I*_1_, *I*_2_, and *I*_3_ flow through NAND strings 1, 2, and 3, respectively. *V*_*C*_ increases to *V*_3_, which equals to (*I*_1_⋅*T*_1_ + *I*_2_⋅*T*_2_ + *I*_2_⋅*T*_2_)/C. Here, for simplicity of description, it is assumed that the weights of cells to which read bias is applied are the same. The VDD and ground limit the voltage of the capacitor. Therefore, the relationship between *V*_C_ and the difference of *I*_EVEN_ and *I*_ODD_ represents a hard-sigmoid function, which is one of the activation functions, as shown in Figure 2C. Note that *V*_*C*_ linearly increases with the difference of *I*_EVEN_ and *I*_ODD_ in a specific current region where the difference of *I*_EVEN_ and *I*_ODD_ ranges from -(C⋅VDD)/(2⋅*T*_read_) to (C⋅VDD)/(2⋅*T*_read_). Here, for simplicity of description, it is assumed that *I*_EVEN_ and *I*_ODD_ are constant during *T*_read_. Therefore, this scheme can process MAC of 4-bit weight and analog input pulse and implement neuron activation in a single input step without any logic operation, significantly reducing the burden of peripheral circuits required for logic operation. The PWM circuits, current mirrors, and capacitors are reused for all synapse layers (equivalently WLs) in a synaptic string, which greatly reduces the area of peripheral circuits. Note that the convolution operation and VMM in multilayer neural networks are the same operations in principle when a 2D convolution kernel is unrolled into a 1D column (Gao et al., 2016). Therefore, the proposed scheme in this work can be applied to the implementation of convolutional neural networks.

## Results

### Measurement Results of NAND Flash Cells

**FIGURE 3:**
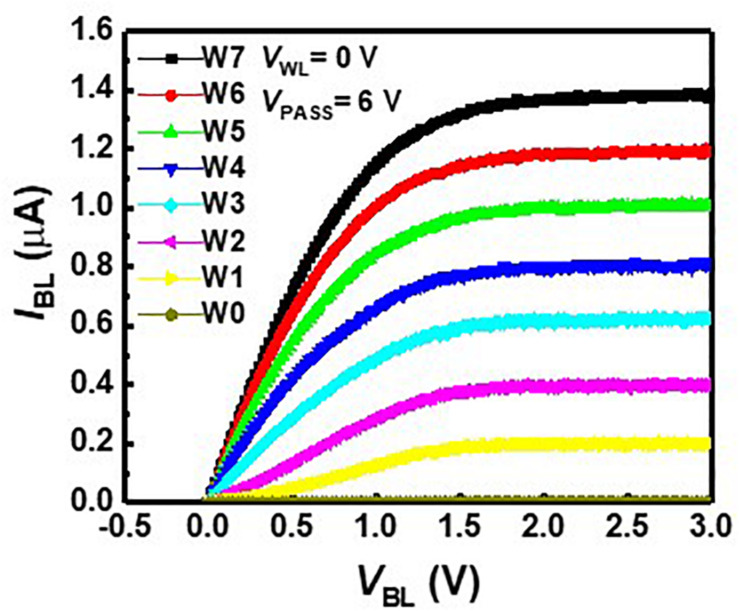
*I*_*BL*_–*V*_*BL*_ characteristics with various weight levels at a *V*_*PASS*_ of 6 V and a *V*_*WL*_ of 0 V.

We measured floating-gate 2D NAND cells fabricated with 26-nm technology. One cell string is composed of 64 cells, including a ground select line transistor, an SSL transistor, and two dummy cells. The channel width and length are 20 and 26 nm, respectively. Figure 3 represents BL current (*I*_BL_) versus BL voltage (*V*_BL_) curves with various weight levels at a *V*_*PASS*_ of 6 V and WL voltage (*V*_WL_) of 0 V. Each cell has eight weight levels giving eight current levels from 0 to 1.4 μA, and the current difference between adjacent current levels is 200 nA. As one synaptic device consists of positive and negative weight cells, the synaptic device has a 4-bit weight. In the neuromorphic system, the IR drop of metal wire causes inaccurate VMM operation, as resistance in metal wire decreases effective voltage imposed on synaptic devices. In addition, the channel resistance of adjacent cells where pass bias is applied also results in inaccurate VMM operation in NAND flash memory. To resolve these problems, NAND cells are operated in the saturation region, eliminating the problem caused by the resistance of the metal wire and the pass cells in the unselected layers. *I*_*BL*_ rarely changes despite the change of *V*_BL_ in the saturation region, as shown in Figure 3, and the minimum output resistance of a NAND cell, which operates at a saturation region, is about 20 MΩ.

**FIGURE 4:**
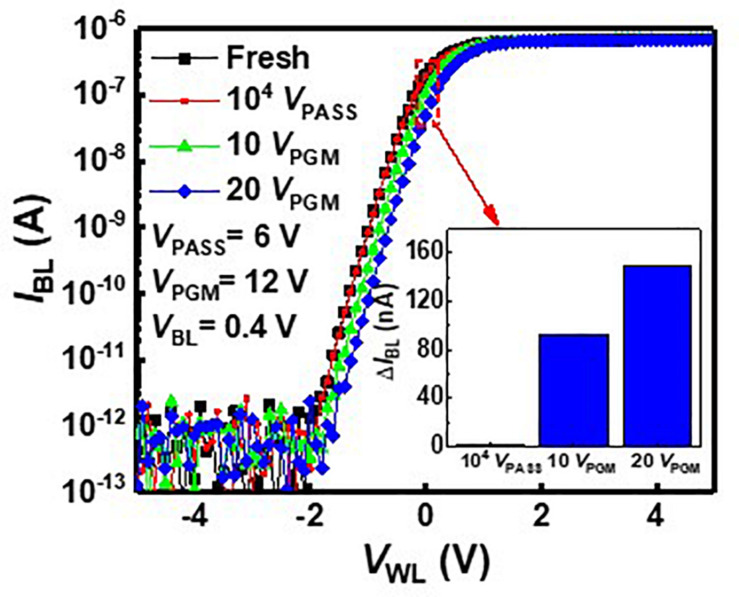
*I*_*BL*_–*V*_*WL*_ characteristics with *V*_*PASS*_ and *V*_*PGM*_.

As *V*_PASS_ is applied to pass cells during the inference process, *V*_PASS_ disturbance needs to be investigated. Figure 4 shows the *I*_BL_–*V*_WL_ curves with *V*_PASS_ disturbance and 12-V program bias (*V*_PGM_). Black square symbols represent the *I*_BL_–*V*_BL_ curve measured in a fresh cell. The red circle symbol represents the *I*_BL_–*V*_BL_ curve after applying *V*_PASS_ of 6 V 10^4^ times to the fresh cell. As these two curves are nearly the same, the effect of *V*_PASS_ is negligible. The curves measured after a pulse with *V*_PGM_ of 12 V is applied to the cell 10 times and 20 times, which are depicted by green triangle symbols and blue diamond symbols, respectively. The inset shows the change of *I*_BL_ (Δ*I*_BL_) after applying 10^4^ *V*_PASS_ (6 V), 10 *V*_PGM_, and 20 *V*_PGM_ pulses. As shown in the inset, the *I*_BL_ shows little variation with 10^4^ *V*_PASS_ pulses compared with 10 *V*_PGM_ pulses.

**FIGURE 5:**
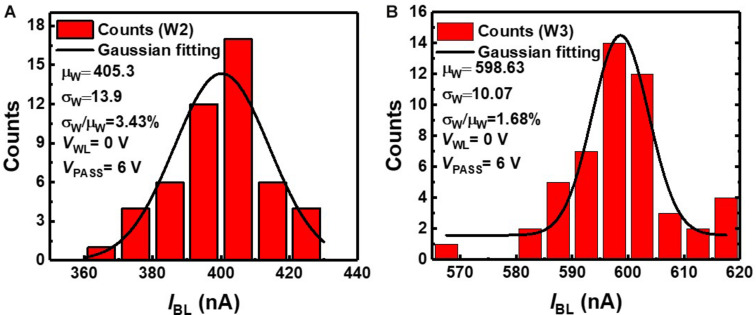
*I*_BL_ distribution of NAND cells in NAND array at **(A)** W2 and **(B)** W3 levels.

We estimate device variation, as it degrades the classification accuracy of neural networks. RVW method is used to match *I*_BL_ of NAND cells in a NAND array to the target current level among eight levels in Figure 3. The weights obtained in off-chip training are transferred to cells by the RVW method, which reiterates the cycle of reading, verifying, and writing threshold voltage of NAND cells. After each *V*_PGM_ pulse is imposed on the NAND cell, the *I*_BL_ of the NAND cell is measured by the *V*_read_ to check if the measured conductance of the cell is outside of the target conductance range. A *V*_PGM_ is imposed on the NAND cell if the conductance is outside of the target conductance range. As this process is repeated, the amplitude of *V*_PGM_ increases. The RVW process ends when the conductance of the cell is within the target conductance range. In this work, ∼40 pulses are applied to fit the current of a synaptic device within the range of target current on average, and amplitude of *V*_PGM_ increases from 11 V with a fixed width of 100 μs. Figure 5 shows the measured *I*_BL_ distribution of second and third weight levels (W2, W3) obtained by the RVW method in the NAND string. To investigate the effect of device variation on neural networks, the largest variation among the eight levels need to be estimated. Among the eight levels, W2 has the largest device variation, and W3 has the smallest device variation. The estimated device variation (σ_*w*_/μ_*w*_) of W2 is 3.43%, and W3 is 1.68% based on the statistical parameters extracted from the measurement data. In this estimation, we assume that the conductance distribution of NAND cells follows a Gaussian distribution (Lee et al., 2019b).

### Pulse Width Modulation Circuit

**FIGURE 6:**
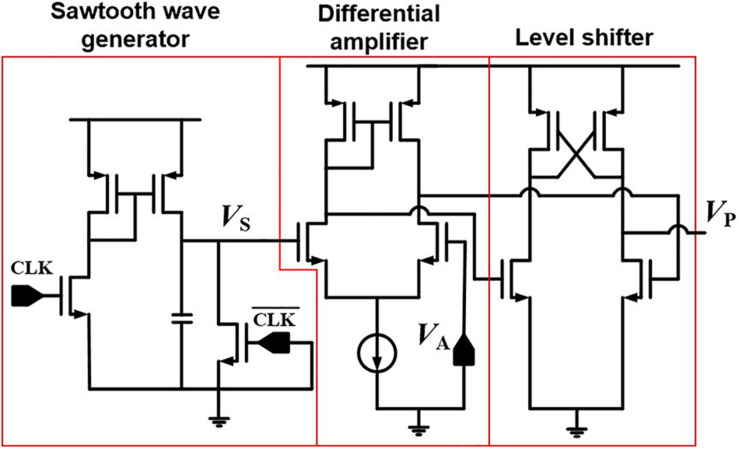
PWM circuit consisting of sawtooth wave generator, differential amplifier, and level shifter.

**FIGURE 7:**
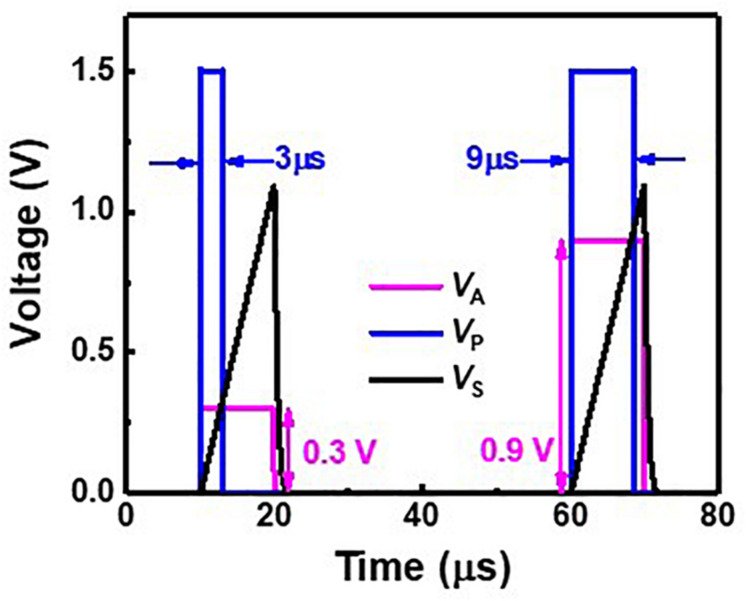
Simulated result of *V*_*A*_, *V*_*S*_, and *V*_*P*_ in PWM circuit.

Figure 6 represents a PWM circuit consisting of a sawtooth generator, a differential amplifier, and a level shifter. The sawtooth generator produces a sawtooth wave (*V*_*S*_). The differential amplifier compares *V*_*S*_ with an analog signal (*V*_*A*_) and amplifies the difference between *V*_*S*_ and *V*_*A*_. The level shifter produces a width-modulated pulse (*V*_*P*_) with a fixed amplitude, and *V*_*P*_ is applied to SSLs of a synaptic string array. Figure 7 shows the simulation results of *V*_*A*_, *V*_*S*_, and *V*_*P*_ in the PWM circuit when *V*_*A*_s are 0.3 and 0.9 V, as an example. The pulse width of the *V*_*P*_ is proportional to the amplitude of the *V*_*A*_. As the amplitude of *V*_*A*_ increases from 0.3 to 0.9 V, the pulse width of *V*_*P*_ increases from 3 to 9 μs.

### Evaluation of Quantized Neural Networks

**FIGURE 8:**
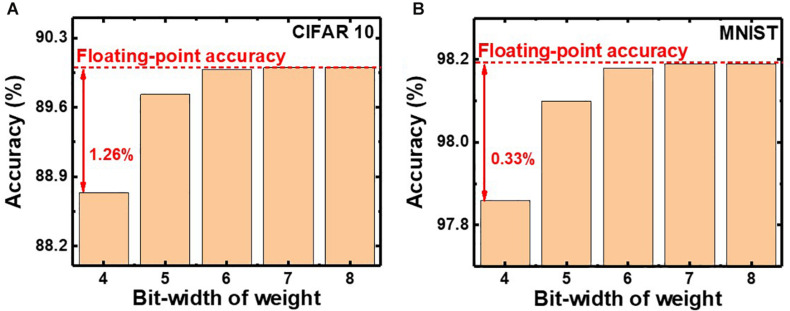
Simulated classification accuracy with respect to the bit-width of weight using PTQ for **(A)** CIFAR10 and **(B)** MNIST images.

In QNNs, the weight can be quantized during or after training. PTQ means that training the DNNs with high-precision floating-point weight without quantization during training. After the training process, PTQ quantizes the pretrained weight at the inference stage. On the other hand, QT performs quantizing the weights during the training process and training a DNN model with quantized weights during forward and backward propagations (Li et al., 2017a,b; Choi et al., 2019). We investigate the effect of QT that involves quantization during the training process on the inference accuracy. Figures 8A,B show simulated classification accuracies of QNN using PTQ for CIFAR10 and MNIST, respectively. Classification accuracies decrease by 0.33 and 1.26% with PTQ for MNIST and CIFAR10 images, respectively, compared with those obtained from neural networks having floating-point weight, as the bit-width of weight decreases to 4. Therefore, the PTQ scheme significantly decreases inference accuracy with 4-bit weight.

**FIGURE 9:**
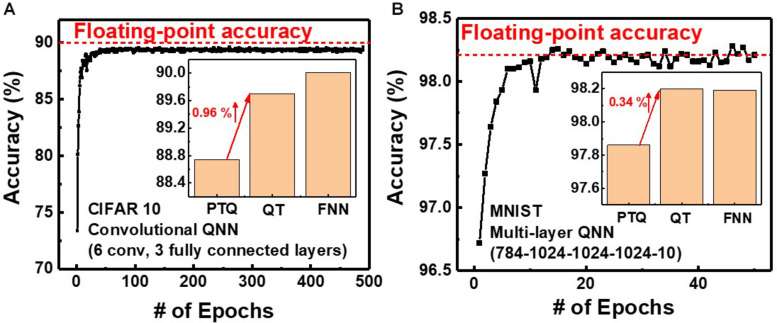
Simulated classification accuracy with QT for **(A)** CIFAR10 and **(B)** MNIST images.

To decrease the degradation of classification accuracy, we adopt QT, which is an algorithm that involves fine-tuning optimized for QNN. Figure 9 shows the simulated classification accuracy of neural networks using QT. QT increases classification accuracies by 0.34 and 0.96% for MNIST and CIFAR10, respectively, compared with those for PTQ. The classification accuracies using QT for MNIST and CIFAR10 are 98.2 and 89.7%, respectively, which are comparable with those obtained in neural networks having floating-point weight (FNN), as shown in the inset. Therefore, by adopting QT, the neuromorphic system utilizing NAND flash memory weighting 4-bit can achieve high inference accuracy. The power efficiency of the synaptic device is estimated from the distribution of synaptic weights in QNN. The average power consumed in a synaptic device per neural computation is estimated to be 0.15 μW for multilayer neural networks consisting of five layers (784–1024–1024–1024–10). The power consumption of the synaptic device can be reduced by adopting a thin (∼3 nm) body (Lue et al., 2019) or pruning the neural networks (Lee et al., 2020). Note that, in this work, we use a 4-bit weight because a 4-bit weight can achieve higher accuracy than binary weight and achieve comparable accuracy compared with a 6-bit weight (Hubara et al., 2017). If a synaptic device has a 5-bit conductance level to implement a 6-bit weight, more time and energy are required in the RVW process for weight transfer.

**FIGURE 10:**
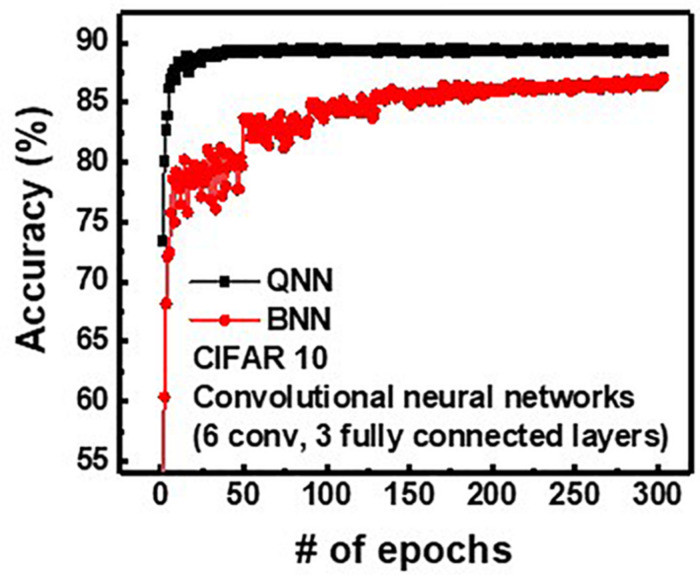
Simulated classification accuracy of QNN and BNN for CIFAR10 images.

To investigate the effect of weight and input precision on the classification accuracy of the neural networks, QNN, having 4-bit weight and analog input, is compared with binary neural networks (BNN) having 1-bit weight and 1-bit input. Figure 10 shows the inference accuracy of QNN and BNN for CIFAR10 with convolution neural networks having three fully connected layers and six convolution layers. Note that, as bit-width of weight and input in QNN decreases, the classification accuracy decreases (Hubara et al., 2017). It is because the quantization of weights and inputs results in a weighted sum error. In addition, the reduction of bit-width of quantization increases the weighted sum error, which decreases classification accuracy. The final classification accuracies are 89.38 and 87.1% for QNN and BNN, respectively. Therefore, the proposed operation scheme can implement QNN with higher inference accuracy compared with BNN (Lee et al., 2019a).

### Effect of Device Non-ideality

**FIGURE 11:**
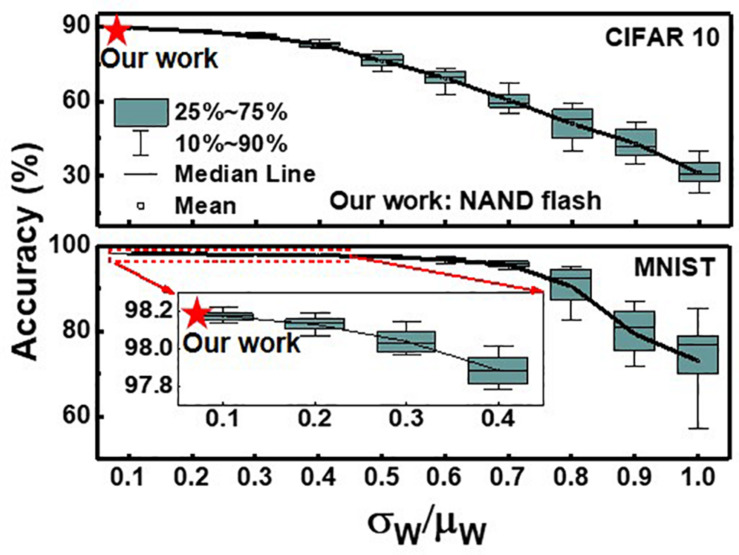
Effect of device variation (σ_*w*_/μ_*w*_) on simulated classification accuracy of QNN for CIFAR 10 and MNIST images. Red star represents the accuracy when the largest variation obtained in this work is applied.

Figure 11 shows the effect of device variation (σ_*w*_/μ_*w*_) on simulated classification accuracy of QNN for CIFAR10 and MNIST images. The simulation is executed 20 times at each σ_*w*_/μ_*w*_, assuming a Gaussian distribution (Lee et al., 2019b). The classification accuracy decreases as the device variation increases. In this work, the largest device variation among eight levels is 3.43% (W2), so it is used to estimate the classification accuracy. As the device variation (σ_*w*_/μ_*w*_) of our work is sufficiently low, the inference accuracies decrease by less than 0.16 and 0.24% for the MNIST and CIFAR 10 images, respectively, compared with accuracy with no variation. To reduce the variation in the conductance of synaptic devices, it is necessary to reduce the target current range set in the control circuits of the RVW method. However, this increases the number of pulses applied to devices, which increases energy and time consumption in the RVW process. Therefore, it is necessary to set the optimized target current range in RVW, taking into account the degree of conductance variation and the energy and time consumed in the RVW process. The variation obtained in this work is less than 3.43%, which is sufficiently low to achieve comparable accuracy compared with that with no variation.

**FIGURE 12:**
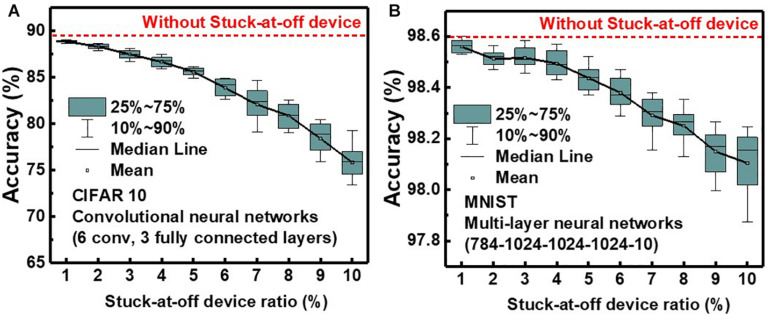
Effect of stuck-at-off device ratio on simulated classification accuracy of QNN for **(A)** CIFAR 10 and **(B)** MNIST images.

Figure 12 shows the effect of the stuck-at-off device ratio on simulated classification accuracy of QNN for CIFAR10 and MNIST images. The simulation is executed 20 times at each ratio, and the classification accuracy decreases as the ratio of stuck-at-off device increases. The classification accuracies decrease by 13.5 and 0.5% for CIFAR10 and MNIST, respectively, as the stuck-at-off device ratio increases to 10%. To reduce degradation of classification accuracy due to the stuck-at-off device below 1% for CIFAR10, the ratio of the stuck-at-off device needs to be below 2%. NAND flash memory is currently a mass-produced technology, and the ratio of stuck-at-off cells is estimated to be less than 1%.

## Discussion

### Comparison of Input Pulse Schemes

To implement VMM in a neuromorphic system, the intensity of the input signal in the DNN algorithm can be represented by the amplitude or width of the input pulse. However, the amplitude modulation scheme causes an error in VMM because the *I-V* characteristics of synaptic devices are non-linear (Kim T. et al., 2017). To resolve this problem, a previous study reported an input pulse mapping scheme using an inverse function generator that handles the non-linearity of *I-V* characteristics (Kim T. et al., 2017). This solves the non-linearity problem, but the VMM can still be inaccurate due to unwanted voltage drop across the parasitic resistance of the pass cells or metal wire. As described earlier, the amplitude modulation scheme has limitations in realizing accurate VMM operation but can reduce latency compared with the width modulation scheme.

On the other hand, the width modulation scheme can eliminate the effect of parasitic resistance by operating synaptic devices in the saturation region of *I-V* characteristics. This scheme may have a longer latency than the amplitude modulation scheme but enables accurate VMM. The width modulation scheme requires a PWM circuit to convert the intensity of the input to the width of the input pulse, which increases the burden on the peripheral circuit. Because the amplitude modulation scheme requires an inverse function generator that requires an operational amplifier, it also increases the burden on the peripheral circuit (Kim T. et al., 2017).

### Comparison With Prior Works

In prior studies, our group has reported neuromorphic architectures that use NAND flash memory cells as binary synapses performing XNOR operation in BNNs (Lee et al., 2019a) and synaptic devices in on-chip training (Lee et al., 2018). In those studies, output current for each neuron is sequentially generated each time *V*_read_ is imposed on a selected WL. However, in this work, all outputs of neurons in a neuron layer are generated in a single input pulse. In addition, in the previous study of Lee et al. (2018), the conductance of synaptic devices is changed by applying an identical pulse to the synaptic device in on-chip learning. In this study, the conductance of synapse is tuned by the RVW method in off-chip learning. In Lee et al. (2019a), binary synaptic architecture capable of XNOR operation digitally was reported. However, this work proposes the VMM of multi-bit input and multi-bit weight in an analog fashion, significantly decreasing the burden of neuron circuits compared with the scheme of digital fashion.

A design scheme of synaptic architecture using NAND flash memory for performing MAC with multi-bit weight and multi-bit input has been proposed in Lue et al. (2019). In this scheme, lots of binary cells and BLs are utilized to represent a multilevel weight and a multilevel input, respectively, resulting in a substantial disadvantage in terms of synapse density (Lue et al., 2019). Furthermore, “shifter and adder” design is utilized to generate multilevel MAC, resulting in lots of burden in peripheral circuits (Lue et al., 2019). On the other hand, the proposed scheme in this work uses two NAND cells as one synaptic device and utilizes the PWM circuit to represent multi-bit input, which significantly increases the density of synaptic devices. Furthermore, the VMM can be performed in a pulse step using the proposed scheme in this work, greatly reducing the CMOS overhead in peripheral circuits compared with the “shifter and adder” design.

## Conclusion

We have proposed a novel operating method and architecture for neuromorphic computing using PWM in the NAND flash memory architecture and evaluated its performance. The proposed operation scheme is well fitted to conventional NAND flash memory to implement QNNs with width-modulated input pulse and 4-bit weight. In addition, VMM of analog input and 4-bit weight can be implemented with a single pulse without additional logic operation. By utilizing a RVW scheme, eight conductance levels from 0 to 1.4 μA were demonstrated with a device variation of less than 3.43%. QT increases accuracies by 0.34 and 0.96% for MNIST and CIFAR10 images, respectively, compared with PTQ. Sufficiently low device variation (3.43%) of NAND cells results in high inference accuracy. Finally, the proposed operation scheme in this work can implement high-density, highly robust, and highly efficient neuromorphic systems using NAND flash memory architecture.

## Data Availability Statement

The raw data supporting the conclusion of this article will be made available by the authors, without undue reservation.

## Author Contributions

S-TL and J-HL conceived and designed the experiments and wrote the manuscript. S-TL performed the simulation for MNIST and CIFAR10 classification, theoretical analyses, and measured device characteristics. All authors discussed the results and commented on the manuscript.

## Conflict of Interest

The authors declare that the research was conducted in the absence of any commercial or financial relationships that could be construed as a potential conflict of interest.

## References

[B1] Andri R. Cavigelli L. Rossi D. Benini L. (2018). “YodaNN: an architecture for ultralow power binary-weight CNN acceleration,” in *Proceedings of the IEEE Transactions on Computer-Aided Design of Integrated Circuits and Systems*, Piscataway, NJ 10.1109/TCAD.2017.2682138

[B2] Choi J. Venkataramani S. Srinivasan V. Gopalakrishnan K. Wang Z. Chuang P. (2019). “Accurate and efficient 2-bit quantized neural networks,” in *Proceedings of the 2nd SysML Conference*, Boston, FL.

[B3] Gao L. Chen P.-Y. Shimeng Y. (2016). Demonstration of convolution kernel operation on resistive cross-point array. *IEEE Electron Dev. Lett.* 37 870–873. 10.1109/led.2016.2573140

[B4] Guan Y. Ohsawa T. (2019). “Co-design of DNN model optimization for binary ReRAM array in-memory processing,” in *Proceedings of the 2019 IEEE 11th International Memory Workshop (IMW)*, Monterey, CA 10.1109/IMW.2019.8739722

[B5] Hubara I. Courbariaux M. Soudry D. El-Yaniv R. Bengio Y. (2017). Quantized neural networks: Training neural networks with low precision weights and activations. *J. Mach. Learn. Res.* 18 6869–6898.

[B6] Huh H. Cho W. Lee J. Noh Y. Park Y. Ok S. (2020). “A 1Tb 4b/Cell 96-stacked-WL 3D NAND flash memory with 30MB/s program throughput using peripheral circuit under memory cell array technique,” in *Proceedings of the IEEE International Solid-State Circuits Conference-(ISSCC)*, San Francisco, CA 10.1109/ISSCC19947.2020.9063117

[B7] Jackson B. L. Rajendran B. Corrado G. S. Breitwisch M. Burr G. W. Cheek R. (2013). Nanoscale electronic synapses using phase change devices. *ACM J. Emerg. Technol. Comput. Syst.* 9 1–20. 10.1201/9780367808624-1

[B8] Kang D. Kim M. Jeon S. C. Jung W. Park J. Choo G. (2019). “13.4 A 512Gb 3-bit/Cell 3D 6 th-Generation V-NAND flash memory with 82MB/s write throughput and 1.2 Gb/s interface,” in *Proceedings of the 2019 IEEE International Solid-State Circuits Conference-(ISSCC)*, New York, NY 10.1109/ISSCC.2019.8662493

[B9] Khan S. H. Hayat M. Porikli F. (2019). Regularization of deep neural networks with spectral dropout. *Neural Netw.* 110 82–90. 10.1016/j.neunet.2018.09.009 30504041

[B10] Kim H. Hwang S. Park L. Park B.-G. (2017). Silicon synaptic transistor for hardware-based spiking neural network and neuromorphic system. *Nanotechnology* 28:40. 10.1088/1361-6528/aa86f8 28820141

[B11] Kim T. Kim H. Kim J. Kim J. J. (2017). Input voltage mapping optimized for resistive memory-based deep neural network hardware. *IEEE Electron Dev. Lett.* 38 1228–1231. 10.1109/led.2017.2730959

[B12] Lee S. T. Kim H. Bae J. Yoo H. Choi N. Kwon D. (2019a). “High-density and highly-reliable binary neural networks using NAND flash memory cells as synaptic devices,” in *Proceedings of the 2019 IEEE Int. Electron Devices Meeting (IEDM)*, San Francisco, CA 10.1109/IEDM19573.2019.8993478

[B13] Lee S. T. Lim S. Choi N. Bae J. Kwon D. Park B. (2019b). Operation scheme of multi-layer neural networks using NAND flash memory as high-density synaptic devices. *IEEE J. Electron Dev. Soc.* 7 1085–1093. 10.1109/jeds.2019.2947316

[B14] Lee S. T. Lim S. Bae J. H. Kwon D. Kim H. S. Park B. G. (2020). Pruning for hardware-based deep spiking neural networks using gated schottky diode as synaptic devices. *J. Nanosci. Nanotechnol.* 20 6603–6608. 10.1166/jnn.2020.18772 32604482

[B15] Lee S. T. Lim S. Choi N. Bae J. H. Kim C. H. Lee S. (2018). “Neuromorphic technology based on charge storage memory devices,” in *Proceedings of the IEEE Symposium on VLSI Technology*, Honolulu, HI 10.1109/VLSIT.2018.8510667

[B16] Li H. De S. Xu Z. Studer C. Same H. Goldstein T. (2017a). “Towards a deeper understanding of training quantized neural networks,” in *Proceedings of the ICML 2017 Workshop on Principled Approaches to Deep Learning (PADL)*, Sydney.

[B17] Li H. De S. Xu Z. Studer C. Samet H. Goldstein T. (2017b). “Training quantized nets: a deeper understanding,” in *Proceedings of Advances in Neural Information Processing Systems*, 5811–5821.

[B18] Lue H. T. Hsu P. K. Wei M. L. Yeh T. H. Du P. Y. Chen W. C. (2019). “Optimal design methods to transform 3D NAND flash into a high-density, high-bandwidth and low-power nonvolatile computing in memory (nvCIM) accelerator for deep-learning neural networks (DNN),” in *Proceedings of the International Electron Device Meeting (IEDM)*, San Francisco, CA 10.1109/IEDM19573.2019.8993652

[B19] Nishani E. Cico B. (2017). “Computer vision approaches based on deep learning and neural networks: Deep neural networks for video analysis of human pose estimation,” in *Proceedings of the 2017 6th Mediterranean Conference on Embedded Computing (MECO)*, Bar 10.1109/MECO.2017.7977207

[B20] Park S. Sheri A. Kim J. Noh J. Jang J. Jeon M. (2013). “Neuromorphic speech systems using advanced ReRAM-based synapse,” in *Proceedings of the 2013 IEEE International Electron Devices Meeting*, Washington, DC 10.1109/IEDM.2013.6724692

[B21] Sainath T. N. Weiss R. J. Wilson K. W. Li B. Variani E. Bacchiani M. (2017). “Multichannel signal processing with deep neural networks for automatic speech recognition,” in *Proceedings of the IEEE/ACM Transactions on Audio, Speech, and Language Processing*, Piscataway, NJ 10.1109/TASLP.2017.2672401

[B22] Scardapane S. Comminiello D. Hussain A. Uncini A. (2017). Group sparse regularization for deep neural networks. *Neurocomputing* 241 81–89. 10.1016/j.neucom.2017.02.029

[B23] Sun X. Peng X. Chen P.-Y. Liu R. Seo J.-S. Yu S. (2018). “Fully parallel RRAM synaptic array for implementing binary neural network with (+ 1, − 1) weights and (+ 1, 0) neurons,” in *Proceedings of the 2018 23rd Asia and South Pacific Design Automation Conference (ASP-DAC)*, Jeju 10.1109/ASPDAC.2018.8297384

[B24] Suri M. Bichler O. Querlioz D. Cueto O. Perniola L. Sousa V. (2011). “Phase change memory as synapse for ultra-dense neuromorphic systems: application to complex visual pattern extraction,” in *Proceedings of the 2011 International Electron Devices Meeting*, Washington, DC 10.1109/IEDM.2011.6131488

[B25] Tang T. Xia L. Li B. Wang Y. Yang H. (2017). “Binary convolutional neural network on RRAM,” in *Proceedings of the 2017 22nd Asia and South Pacific Design Automation Conference (ASP-DAC)*, Chiba 10.1109/ASPDAC.2017.7858419

[B26] Truong L. Barik R. Totoni E. Liu H. Markley C. Fox A. (2016). “. Latte: a language, compiler, and runtime for elegant and efficient deep neural networks,” in *Proceedings of the 37th ACM SIGPLAN Conference on Programming Language Design and Implementation*, New York, NY 10.1145/2908080.2908105

[B27] Wang C. Feng D. Tong W. Liu J. Wu B. Zhao W. (2020). “Improving write performance on cross-point RRAM arrays by leveraging multidimensional non-uniformity of cell effective voltage,” in *Proceedings of the IEEE Transactions on Computers*, Piscataway, NJ 10.1109/TC.2020.2990884

[B28] Woo J. Yu S. (2019). “Impact of selector devices in analog RRAM-based crossbar arrays for inference and training of neuromorphic system,” in *Proceedings of the IEEE Transactions on Very Large Scale Integration (VLSI) Systems*, London 10.1109/TVLSI.2019.2917764

[B29] Yamashita R. Magia S. Higuchi T. Yoneya K. Yamamura T. Mizukoshi H. (2017). “A 512gb 3b/cell flash memory on 64-word-line-layer bics technology,” in *Proceeding of the 2017 IEEE International Solid-State Circuits Conference (ISSCC)*, San Francisco, CA 10.1109/ISSCC.2017.7870328

[B30] Yu S. Sun X. Peng X. Huang S. (2020). “Compute-in-memory with emerging nonvolatile-memories: challenges and prospects,” in *Proceedings of the 2020 IEEE Custom Integrated Circuits Conference (CICC)*, Boston, MA 10.1109/CICC48029.2020.9075887

[B31] Zhou Z. Huang P. Xiang Y. C. Shen W. S. Zhao Y. D. Feng Y. L. (2018). “A new hardware implementation approach of BNNs based on nonlinear 2T2R synaptic cell,” in *Proceedings of the IEEE International Electron Devices Meeting (IEDM)*, San Francisco, CA 10.1109/IEDM.2018.8614642

